# Case report: Malignant proliferating trichilemmal tumor of the thumb

**DOI:** 10.3389/fonc.2022.1005206

**Published:** 2022-10-27

**Authors:** Guojie Wang, Xuan Zhou, Junqi Luo, Qiyi Hu, Jie Zhang

**Affiliations:** ^1^ Department of Radiology, Fifth Affiliated Hospital of Sun Yat-sen University, Zhuhai, China; ^2^ Department of Pathology, Fifth Affiliated Hospital of Sun Yat-sen University, Zhuhai, China; ^3^ Department of Nuclear Medicine, Fifth Affiliated Hospital of Sun Yat-sen University, Zhuhai, China; ^4^ Department of Radiology, Zhuhai People’s Hospital, Zhuhai, China

**Keywords:** malignant proliferating trichilemmal tumor, finger, PET/CT, 18-FDG, pathology

## Abstract

Proliferating trichilemmal tumor is a very rare benign tumor that has the potential to transition into a malignant tumor. PTT most commonly affects the scalps of women above 60 years old and is frequently misdiagnosed due to its rarity. Herein, we present a case of a 68-year-old man with a malignant proliferating trichilemmal tumor on his right thumb. X-ray image showed a soft tissue mass on his thumb accompanied by bone destruction, while ^18^F-FDG PET revealed a hypermetabolic mass in the first index with axillary lymph node metastasis.

## Introduction

Proliferating trichilemmal tumor (PTT) is a benign neoplasia originating from the outer root sheath of hair follicles. PTT generally affects older women in the head and neck region and is often misdiagnosed (1, 2). Previous studies have linked trauma and/or inflammation to PTT (3). PTT are benign tumors that can become cancerous, termed malignant proliferating trichilemmal tumor (MPTT) (1, 3). Here, we present an unusual case of MPTT that was detected on the thumb of an elderly man.

## Case report

A 68-year-old man was diagnosed with a soft tissue mass at the distal end of his right thumb that had been present for 6 months but was overlooked due to the mild symptoms. Two months prior to admission, the lesion showed rapid growth accompanied by persistent dull pain. Physical examination revealed swelling at the distal segment of the thumb, local skin flushing, and slight tenderness.

**Figure 1:**
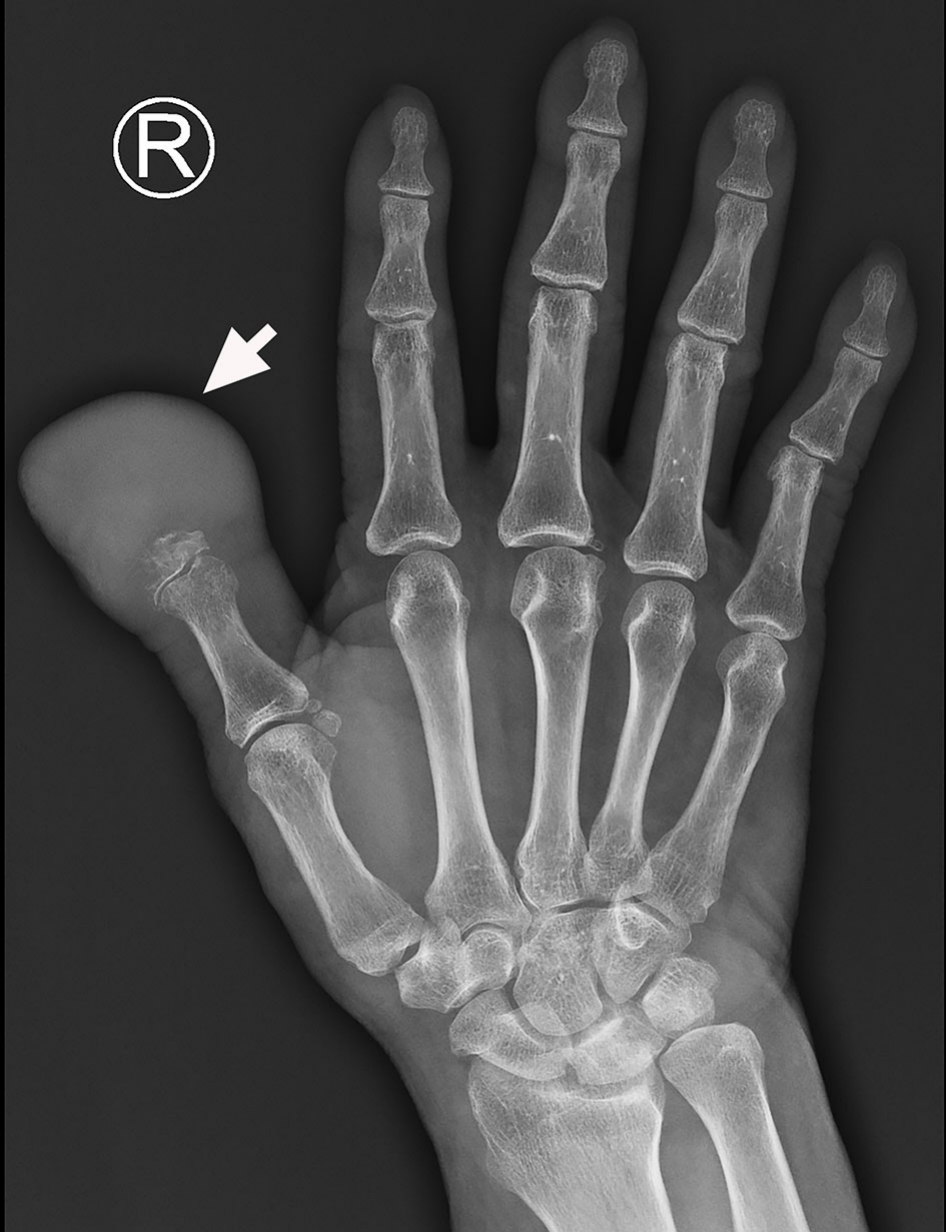
The X-ray image shows a mass at the distal end of the right thumb with marked phalanx destruction (arrow).

**Figure 2:**
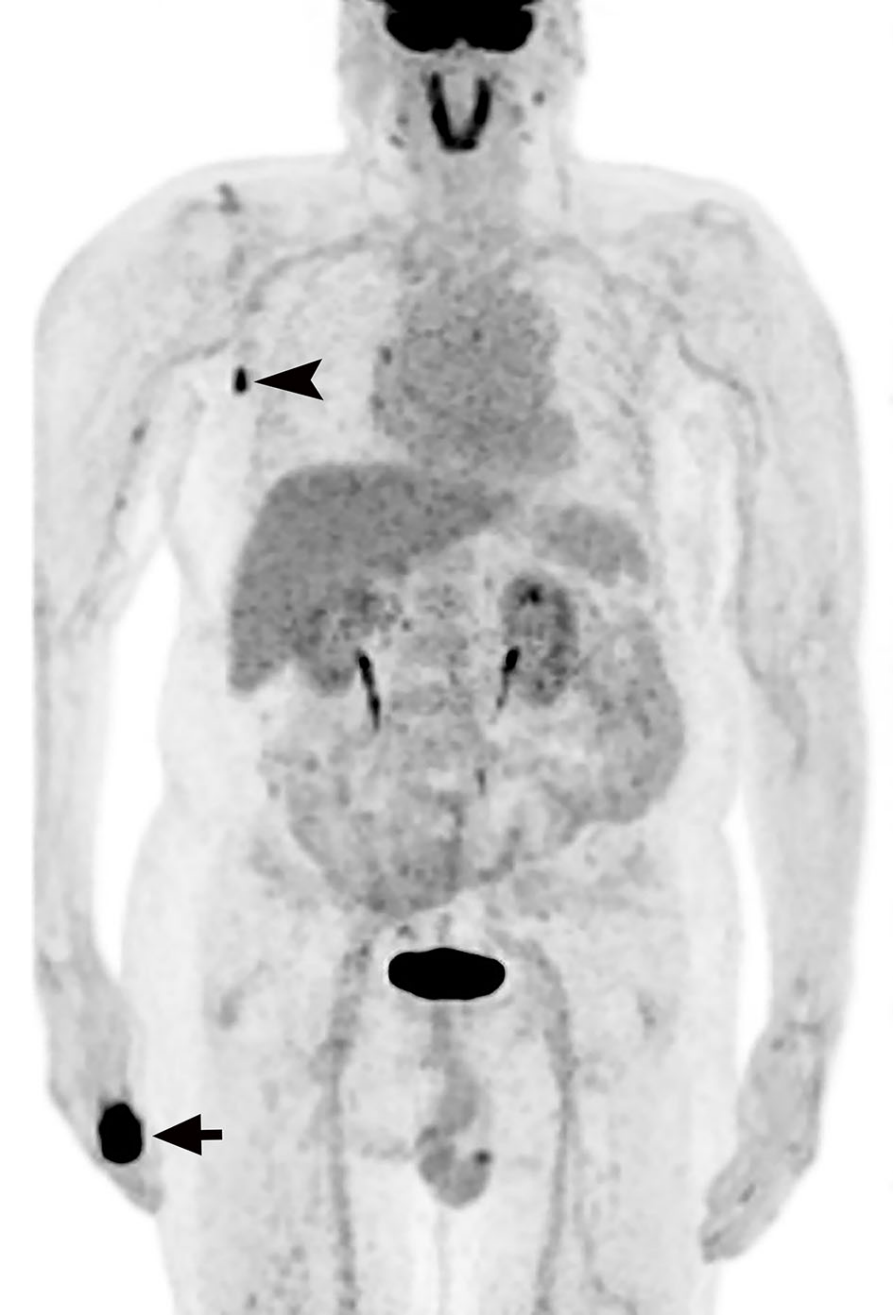
^18^F-FDG PET reveals intense areas of ^18^F-FDG uptake in the right thumb(arrow) and axillary lymph nodes (arrowhead).

X-ray imaging identified a mass at the distal part of the thumb with bone destruction (**Figure 1**). In addition, ^18^F-FDG PET/CT examination revealed a significantly high metabolic activity in the thumb and right axillary lymph nodes (**Figure 2**).

**Figure 3:**
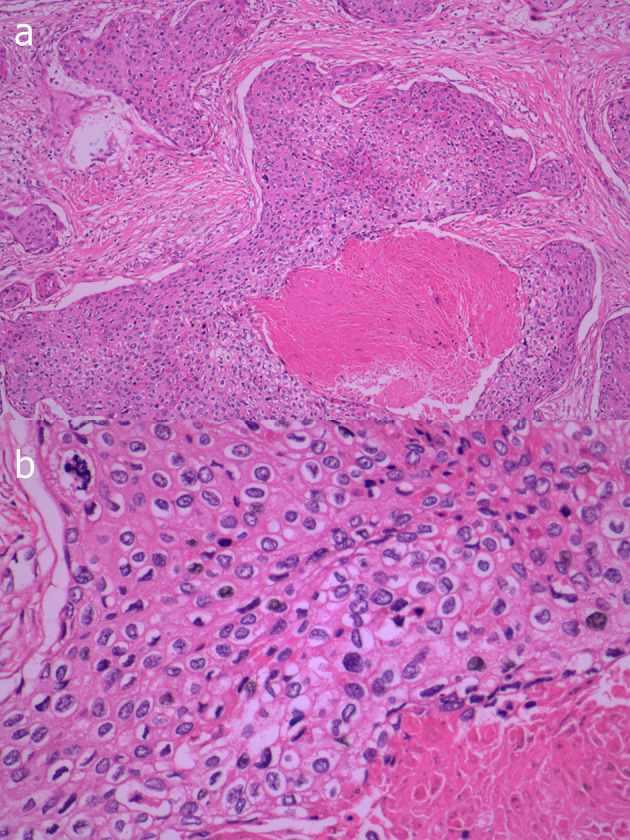
Photomicrograph shows many clumps composed of epithelial cells under the skin, the clumps are lobulated and the marginal cells are distributed in a palisade shape, with obvious keratinization in the center, but lacking a granular layer (**A**, HE×100). In the outer cells, there are obvious cytological atypia and nuclear pleomorphism (**B**, HE×400).

Eventually, we surgically excised the lesion, which was approximately 5.5 cm × 3.5 cm × 3.0 cm in size. Light microscopy revealed that there are many clumps composed of epithelial cells under the skin. The clumps are lobulated, the outer cells are arranged in a palisade shape, and there is trichilemmal keratinization in the center. Between outer cells and central trichilemmal keratinization, there are no granular layers. There are obvious cytological atypia and nuclear pleomorphism in the outer cells (**Figure 3**). Immunohistochemistry results were positive for P63, P40, CK14, and EMA, while the tumor cells were negative for CD34.

Taken together, the above findings support the diagnosis of MPTT. A needle biopsy performed on the right axillary lymph node confirmed the presence of metastatic MPTT. Adjuvant chemotherapy was planned after surgery, and the patient was discharged. After surgical resection, he was followed for 6 months, and there was no report of recurrence.

## Discussion

PTT are rare conditions that mainly occur in women above 60 years of age. Approximately 90% of cases occur on the scalp, with the remaining 10% located in regions such as the back, wrist, nose, and pudenda, to name a few (1). Even less common is PTT of the finger, which has been reported in the literature in only four cases. All four lesions were located in the subungual region (4–7). The researchers hypothesized that the subungual PTT was derived from the nail matrix, which could keratinize to a trichilemmal organization (5). PTT may also result from follicular units containing nail bed epithelium remaining in nails (8).

To the best of our knowledge, MPTT metastases have only been studied with ^18^F-FDG PET in two patients previously (9, 10). In one case, ^18^F-FDG PET was used to assess lymph node metastases caused by recurrent scalp MPTT (10). The other case underwent ^18^F-FDG PET/CT to investigate lymph node metastases of a shoulder MPTT (9). MPTT intensified glucose avidity was present in all cases, suggesting that ^18^F-FDG PET may be useful in the workup of MPTT patients, especially in cases where the tumor’s malignancy potential is not clear.

The pathology of PTT is characterized by trichilemmal keratinization without granular layers. Trichilemmal Cysts, PTT, and MPTT are tumors with differentiation of hair follicles, which have a continuous pedigree from benign to malignant, one end of the tumor is a cystic tumor with a clear boundary, and the other end has malignant characteristics. When the tumor grows rapidly, has invasive growth and distant metastasis, and has a large number of nuclear pleomorphisms and obvious cytological atypia, MPTT should be considered. CD34 is a marker of outer hair root sheath differentiation and is negative in MPTT, which is also the point of differentiation between PTT and MPTT. Desmoplastic trichilemmomas are mostly found on the face, and similar to the trichilemmal tumor, it is characterized by subcutaneous tumor lobules and trichilemmal keratinization, and CD34 is positive. Furthermore, desmoplastic trichilemmomas have strips of epithelial cells growing at the edges (11). Trichilemmal keratinization is not seen in squamous cell carcinoma. Based on this, PTT can be differentiated from squamous cell carcinoma.

In conclusion, this is a rare case of MPTT in the thumb, and ^18^F-FDG PET was useful in confirming axillary lymph node involvement in this rare neoplasia.

## Data availability statement

The raw data supporting the conclusions of this article will be made available by the authors, without undue reservation.

## Ethics statement

Written informed consent was obtained from the individual(s) for the publication of any potentially identifiable images or data included in this article.

## Author contributions

GW, XZ, and JZ: manuscript writing. XZ: pathological review. GW, XZ, JL, QH, and JZ: manuscript revision. All authors contributed to the article and approved the submitted version.

## Funding

This work was supported by the Science and Technology Program of Zhuhai (2220004000244) and the Medical Scientific Research Foundation of Guangdong Province(A2021449).

## Conflict of interest

The authors declare that the research was conducted in the absence of any commercial or financial relationships that could be construed as a potential conflict of interest.

## Publisher’s note

All claims expressed in this article are solely those of the authors and do not necessarily represent those of their affiliated organizations, or those of the publisher, the editors and the reviewers. Any product that may be evaluated in this article, or claim that may be made by its manufacturer, is not guaranteed or endorsed by the publisher.
